# Transition in action: toward a social theory of the governance of transitions

**DOI:** 10.3389/fsoc.2024.1206050

**Published:** 2024-09-18

**Authors:** Marco Billi, Cristina Zurbriggen, Anahí Urquiza, Angel Allendes

**Affiliations:** ^1^Center for Climate and Resilience Research, University of Chile, Santiago, Chile; ^2^Faculty of Agricultural Sciences, University of Chile, Santiago, Chile; ^3^Systemic Transdisciplinary Research Hub NEST-r3, Santiago, Chile; ^4^South American Institute for Resilience and Sustainability Studies (SARAS), Maldonado, Uruguay; ^5^Faculty of Social Sciences, University of the Republic, Montevideo, Uruguay; ^6^Faculty of Social Sciences, University of Chile, Santiago, Chile

**Keywords:** sustainable transition, Luhmann, socio-technical approach, socio-ecological approach, co-construction methodologies

## Abstract

This article explores how a re-interpretation of the socio-technical, socio-ecological and transition design approaches to transition from the point of view of Niklas Luhmann’s general systems theory of society. The need to understand and promote changes that include a technological and ecological dimension has led to different approaches, such as socio-technical or socio-environmental approaches, to incorporate links with society. While these approaches often include sociological insights, they rarely offer a general understanding of how these are embedded into society. We need a new environmental sociology that helps catalyze change processes with a collectively reorganized society, empowering more radically transformative actions to change the current structures and processes that have led us to where we are today. The article offers a cross-sectional look at the socio-ecological and socio-technical systems literature, specifically for what concerns their understanding of the ‘systems’ in transition and how they can be governed, and re-interpret it from the theoretical lens of the deep sociological knowledge, which refers to the profound understanding of social systems and their dynamics, embedded in Luhmann’s theory of social systems. From here, we suggest the second-order coupling for a sociologically grounded understanding of the interactions that comprise socio-ecological and socio-technical systems, heterogeneous and almost self-organizing assemblies of social, technical, and natural elements and processes. At the same time, third-order couplings are analyzed, focused on governance, relationships between operations, and structures mediated by a deliberate attempt to ensure coherence and coordination against the autonomy and heterogeneity of socio-techno-ecological systems. Therefore, this manuscript offers a deeper conceptual and methodological understanding of socio-techno-ecological couplings and systems in the context of sustainability transformation and gives insights into its governance.

## Introduction

Climate change is one of humanity’s most important social and ecological challenges in the 21st century. By 2030, we should at least halve global emissions to keep warming below 1.5 degrees to avoid integrated catastrophic effects and devastating combined consequences such as mega-droughts, desertification, flooding, and direct heat that could affect millions of citizens [Rockström et al., 2009; Intergubernamental Panel on Climate Change (IPCC), 2022]. However, such emissions continue to rise, and achieving the deep transformations required to make this threshold becomes every day more challenging. Added to this process are the climate injustices suffered by poor and marginalized people (Schmitt et al., 2020). Responding to these challenges would require a complete rebuild of the world energy infrastructure, a comprehensive overhaul of agricultural practices and diet to remove carbon emissions from agriculture, and a series of cultural changes in our lives. Besides, we should do that in just two or three decades.

On the upside, our knowledge of climate challenges has accumulated and accelerated. Research has allowed us to improve climate models continuously, generate projections, and study climate change’s alarming and myriad impacts (Rockström et al., 2009; Armstrong McKay et al., 2022). Likewise, an incipient but growing stream of research on multisystem interactions is beginning to unravel their complexity, such as integrated systems modeling (Steffen et al., 2018) or investigating energy, food, and water nexuses (Correa-Porcel et al., 2021). In addition, the contributions of systemic approaches to the sustainable transition have emphasized the interaction between technological transformations and society, such as the approach related to sociotechnical transitions (Geels and Schot, 2007; Billi et al., 2022) or socioecological resilience studies that affirm that environmental problems do not they can be analyzed or understood outside their social context (Folke, 2016; Folke et al., 2021).

From this literature we have learned that we need a new environmental sociology that helps catalyze these processes of change with a collectively reorganized society empowering more radically transformative actions to change the current structures and processes that have led us to where we are today (Dietz et al., 2020; Klinenberg et al., 2020). It is critical to know why, who, when, and where multisystem interactions occur and advise decision-makers to steer and accelerate them, thus taking advantage of potential synergies and avoiding negative interactions. We need to appeal to the creation of critical, ethical, heuristic, and socio-political competencies to learn to build significant knowledge collectively –a collective intelligence– that allows us to define collectively desirable transition paths. Hence, says Sauvé (2017), in the processes of mobilization for social and political demands, it is possible to take awareness of the collective dimension of action: learning to mutually know ourselves, to work together between protagonists, between members of resistance units or project teams; learn to live the inevitable tensions in groups, to resolve conflicts, to face the challenges, to recognize progress. Learn to debate, discuss, argue, deliberate, communicate.

However, while the literature on these topics offers many avenues to incorporate the social dimensions of ecological and technological transitions problems, often touching central themes and traditions of social science scholarship -including, increasingly, questions of agency and power, framing and narratives, inequality and justice, structure and conditioning, among others-it often lacks a thorough reflection on what the social means, and more specifically, what does it entail to treat the social as a system. This is important, because both bodies of literature explicitly stress the need to overcome the traditional distinction between the social and the material (be that ecological or technical) and to do that by understanding them all to be part of the same system. But without a general theory of society, and particularly of society as a system, these claims cannot shed light on the theoretical and practical implications of attempting to shape a systemic governance of society-nature-technology interaction, nor on the specific challenges that modern societies entail for this task.

Against this, Niklas Luhmann’s social systems theory can contribute as a bridge between the social sciences and the broader systems literature addressing issues related to climate change. In general, systems theories have tended to receive less resonance within the contemporary social sciences than in other fields because of its language and fundamental tenets seemingly at odd with the fundamental interests and concepts that concern many social scientists (Olsson et al., 2015). In particular, while these theories became quite influential in the social sciences during the 1950s and 1960s thanks to the works of Talcott Parsons, these later received many criticisms, partly due to its allegedly politically conservative view and incapability of explaining social change. Part of the current disinterest towards systems theory may be explained by their continued association of it with Parsons’ version, although Luhmann quite famously distances himself from Parsons at the beginning of his work. Since the 80’s, there are a number of attempts to revitalize systems theory within the social sciences: among these attempts to build a systemic theory of society, Luhmann’s theory is definitely the most radical, one of the most prominent, and one of the few ones to build a full general theory of society on these premises.

In particular, in this paper, we want to reflect on the usefulness of employing it as a reading lens to deepen and articulate the insights derived from socio-ecological, and socio-technical systems and transition design literature. This task, of course, is not free of challenges: in particular, articulating social theories from different traditions may lead to potential clashes among their underlying ontological and epistemological assumptions. In this sense, we recognize that Luhmann’s social systems theory is founded on different epistemological and ontological assumptions are different from the other approaches to socio-technical, socio-ecological and transition design that build upon the critical realist philosophy of science, such as those of Geels (2018), Sorrell (2018), and others. This may lead some to argue (see, for instance, Wan, 2011a,b) that attempting to articulate these theories together does not make sense, or in fact, it could even lead to an epistemic fallacy. Wan’s criticisms focus on Luhmann’s exclusive focus on the social construction of reality, reflecting whether this aspect of the theory may make it contradictory to articulating Luhaman’s thinking with approaches grounded on critical realism or other more materialist approaches, which often inspire the theories of transitions and resilience. More specifically, Wan sees Luhmann’s constructivist (i.e., epistemology centered) approach as failing to address appropriately the issues concerning emergence (e.g., the level structure of the world), which would make Luhmann’s theory, in fundamental ways, out of step with contemporary science. However, this does not necessarily account for a complete rebuttal of the theory *per se*: the interdisciplinary ambition and the flexibility and adaptability of Luhmann’s systems theory to integrate different strands of the sociological realm with other layers of research is something that critical realism also shares. However, the challenge is for Luhmann’s scholars to not be content with repeating Luhmann’s arguments or applying them to social research uncritically” (Wan, 2011b, 708).

Indeed, this is the challenge that we want to take here. In this sense, there are several ways in which theories can be put into dialogue one with another, and not all of them need perfect compatibility on their fundamental assumptions. That may be required when one wants to offer a meta-theory or overarching integration of the approaches at hand, but not so much when the idea is only to compare approaches, ot to observe and criticize one from another, or to extract insights from one into another. In fact, the very Geels uses this approach to identify possible points of contact or mutual collaborations between the multi level perspectives and other ontologies on the social (Geels, 2010)^1^. In this very same sense, we here aim to use Luhmann’s theory as an analytical lens or frame^2^ that, precisely because of its different epistemological and ontological background, can shed light on the possible blind spots within socio–technical and socio-ecological approaches. And from that standpoint, can help build different alternatives to re-interpret the key insights of these theories in a way which may arguably help designing strategic actions for systemic change.

With this objective, the document begins in the first section with a cross-section reading on socio-ecological and socio-technical systems theory, and the relevance of a deeper reflection on the nature of these “systems” and how this may condition their governance, also referring to some previous attempts to illuminate these topics. Then, it proceeds to describe Niklas Luhmann’s social systems theory, and elaborates on how it can be used to re-interpret and reframe the concept of the system embedded in the previous literature, and what does it mean for their governance. Subsequently, it moves on to discuss an emerging action-oriented approach, transitional design (Irwin, 2015; Irwin et al., 2020), applying the proposed theoretical lens to clarify its governance challenges and proposed mechanisms. On this basis, it discusses how can these kind of approaches help to develop a more integrated perspective on environmental governance, and understand how the communication processes impact governance dynamics to develop social actions against climate change.

### Sustainability transitions and societal transformation: insights from socio ecological and socio-technical systems theories

Sustainability transitions have become an important academic field within sustainability science, highlighting sociotechnical and socioecological approaches. While there are etymological differences between the terms transition and transformation and, ultimately, different concerns regarding scales, both research communities apply systems thinking, share similar goals, and have grown closer in the recent past (Köhler et al., 2019; Billi et al., 2022).

Socio-technical systems literature identifies a broad array of scholarship brought together by a systemic approach to sustainability-related innovation and technological change (Geels, 2022). A central aim of transition research has been to conceptualize and explain how radical changes can occur in how societal functions are met sustainability transitions field, highlighting its origins in innovation studies and the systemic perspective that underlie the four main theoretical frameworks of transition studies, which are the Multi-Level Perspective (MLP), the Technological Innovation System approach (TIS), Strategic Niche Management (SNM), and Transition Management.

The theoretical strand is most famously represented by the so-called multi-level perspective (MLP), which observes transitions as non-linear processes emerging from the interactions between niches (protected spaces where radical innovations occur), regimes (semi-coherent sets of rules and structures which provide stability to the systems) and landscapes (slowly changing variables and trends influencing socio technical actors but unvarying in the short run) (Geels, 2011).

The central ideas are the regimes select which innovations prosper and which fail, and they foster selected innovation, providing them an ecosystem of innovation in the form of complementary technologies, available investments, favorable regulatory frameworks, cultural values and practices promoting their usage, entrepreneurial willingness and capabilities to adopt them, effective markets, etc. However, regimes can lock in technological (and social) change along specific pathways and cause inertia preventing attempts to shift it away from such pathways since radical innovation steering outside the predetermined momentum of innovation tends to not resonate with incumbent structures and be rejected. However, regimes can themselves be changed when alternative solutions developed in the niches reach a critical mass breaking down the inertia of incumbent structures: when that happens, the regime ‘transitions’ to a new equilibrium, a new attractor, with new sets of rules, markets, practices, policies, scientific and technological paradigms, etc. (Geels and Kemp, 2007; Geels and Schot, 2007; Markard et al., 2020).

Independently of specific models, one common concept is that socio-technical transitions are emergent, i.e., they result from complex and partly predictable (and sometimes even unintentional) interactions between multiple groups (Loorbach et al., 2017). Thus, they cannot be steered at will by public authorities, which should be aware that policy designs may have unpredictable and unintended effects (Rip, 2006; Voß et al., 2009), sometimes becoming the cause of the very problems they aim to solve or generating new problems. This suggests the need of rethinking about the way in which we pursue transformations and their governance.

Another fundamental contribution to transformation studies derives from socioecological system theory. This approach emphasizes that social and ecological systems are tightly interconnected and an alteration in one sub-system likely leads to modifications in the other (Folke, 2016). Starting in the 1970s, this approach incorporates the concept of resilience in order to describe the ability of such systems to absorb the disturbances in their surroundings, combining change with the preservation of the relationships between its components. Although such a concept, which came from engineering and the science of materials, originally was attached a very precise scientific meaning in reference to the time needed to recover from a disturbance (Siang et al., 2013). The complex adaptive system approach (CAS) took an early distance from such a narrow definition in favor of one which would include humans and their actions in the system under study, thus considering interdependencies between ecological and social processes, cross-scale interactions and the ability of such systems to shift within multiple regimes (Holling, 1973; Gunderson and Folke, 2005) or basins of attraction (Walker et al., 2004). Precisely because complex adaptive systems present multiple regimes of stability, the notion of resilience encompasses both the degree to which affectations or modifications in the structure of a system can be triggered by external or internal disturbances (Folke, 2006; Gallopin, 2006), including the identification of given stress thresholds which, if overcome, would determine a non-return point, leading to a radical or catastrophic change in the characteristics of the system (Scheffer et al., 2002), as well as the very ability of the system to change rapidly and seamlessly from one regime of stability to another (Gotts, 2007), reorganizing itself to adapt to its new context (Adger et al., 2011; Clarvis and Allan, 2014). More precisely, then, social ecological resilience acknowledges it as “the capacity of a system to absorb disturbance and reorganize while undergoing change so as to still retain essentially the same function, structure and feedbacks, and therefore identity (Folke, 2016), which encompasses both adaptability (actions that sustain development on current pathways) and transformability (shifting pathways or creating new ones).

The socioecological resilience approach adds a new lens to understand bettercollective actions and their implication on governance regimes, with a focus on understanding.

engineering the resilience of systems at different scales (Lebel et al., 2006; Madni and Jackson, 2009), on be more adaptive to shifting environments (Chaffin and Gunderson, 2016) or to transforma them towards more desirable trajectories (Chaffin et al., 2016). Under this lens, social-ecological resilience teaches about understanding and cultivating the capacities of agents and actors, communities, societies, or cultures, to live and develop with expected and surprising change and diverse development pathways and potential thresholds between them. Hence, it is a forward-looking approach to the capacities of intertwined social-ecological systems to persist, adapt, and transform as part of the Anthropocene biosphere. Transformation involves multiple elements: agency, practices, behaviors, incentives, institutions, beliefs, values, and worldviews. Understanding transformation goes beyond focusing on what may trigger changes, hoping to unravel the capabilities to reduce the resilience of an undesired system and nurture and navigate the emergence of new desired systems; to cope with path dependencies, anticipate shocks and surprises, and shift towards sustainable paths. Accordingly, real world transformations would come through thanks to aligning mutually reinforcing processes within and across multiple levels (Folke et al., 2021).

Some aspects that have both approaches shared are an interdisciplinary inspiration, orientation to complexity, and a holistic outlook. However, both these approaches have not been exempt from criticism. Its critics have lamented its tendency to minimize political values (Meadowcroft, 2005) and demanded a stronger focus on the interactions between multiple and simultaneous changes in different environmental and social contexts (Elmqvist et al., 2019) and on the challenges involved in promoting just transitions (Hughes and Hoffmann, 2020). On top of that, both perspectives rarely offer a general understanding of different fields of society, usually limiting themselves to some sphere of attention (i.e., they act as middle-range theories instead of general theories of society). That also means that each may overlook possible insights or relevant questions about other fields of inquiry.

These criticisms should not be seen as a reason to reject socio-technical or socioecological theories but rather as pointing to the need for a deeper sociological reflection on these theories’ ‘social’ component. In particular, on how specific insights derived from the rich literature can help steer profound socio-cultural transformation and depth, taking advantage of the knowledge accumulated in the broader literature in sociological theory is a real feat to face the complex and urgent transformations we need to manage in the future. It is this exercise, deeply transdisciplinary, which we want to attempt in this paper.

In particular, in this manuscript we would like to focus specifically one particular dimension which, in our view, has been so far insufficiently tackled within both socio ecological and socio-technical systems approaches, and which we believe can be illuminated thanks tto the general theory of society offered by Luhmann’s work. The question we would like to tackle in this paper is the following: by definition, both sociotechnical and socioecological systems are understood as heterogenous entities composed of several types of elements and operations coupled one with another. But then, what is the connective tissue which maintains these elements and operations held together?

While simple, this question in fact has to date mostly evaded the attention of the majority of scholars in the field. Particularly in the case of socio-technical systems, the definition of the idea of the socio-technical, as much in terms of the difference between the ‘social’ and ‘technical’ components ot and in terms of what brings together, is often taken for granted, and not thoroughly problematized (on this respect, see, for instance).

Should we take it that the boundaries and dyamics of socio-technical or socio-ecological system are just an analytical tool, a dispositif built by the scientist attributing a contingent correlation of otherwise unconnected processes and successions of events to an underlying, latent structure that in fact we are not even sure is there?

If the fact that practices, rules, policies, markets, and technologies seem to move together were only to depend y the contingent choice of the analyst, then it would lose all meaning to speak of a system, and to analyze it ‘as’ a system, and even less, to try and ‘manage’ it as a system such as both Transition Management and Adaptive Governance approaches do, for example. We need, then, a more robust definition of what makes that system a system, and what does it then mean to attempt to govern such a system.^3^ Ours is not of course, the first effort in this direction: among the most prominent previous works on this matter it is due to recognize, at least, John Mingens’ critical realist systems theory, Mario Bunge’s emergent materialist account of social systems, or Edwin Hutchins’ account of distributed cognitive systems, among others. While we cannot discuss them into detail here, all of these works offer illuminating and deeply inspiring contributions to better understand emerging systems and their dynamics.

In this paper, we hope to offer an additional avenue for problematizing and approaching this problem from Luhmann’s perspective, which we think offers very illuminating insights which can complement existing efforts in the area. In particular, through Luhmann’s theory, we hope to bring further light on how these interactions manifest in the concrete practice of transformation, in the different and multilayered territorial contexts where changes are made and promoted, and where governance challenges out. Given the incredibly complex, diverse, multidimensional, and multidisciplinary nature of environmental problems, there is a need for ways to help translate and articulate findings in different domains of social knowledge and thus advance toward a genuinely transdisciplinary outlook on the social dynamics of environmental issues, environment oriented actions, and policies. As mentioned above, there is then, in principle, an opportunity^4^ to use Luhmann’s sociological theory as a second-order lens or ‘frame’ to observe and ground, empower, and articulate different middle-rangesystemic approaches illuminating or proposing solutions for the environmental challenges of our society.

### Niklas Luhmann’s social systems theory

Luhmann’s theoretical apparatus is one of the most ambitious and comprehensive approaches to understanding contemporary world society, offering a complex, multilayered and self-reflexive theoretical understanding of society. According to Luhmann (1999, 2007, 1992), society must be understood not as the sum of individual persons or territorial entities, but as a self-referential system, constitutive of meaning, whose basic operation is communication. Drawing from previous advancements achieved in the field of second-order cybernetics (von Foerster, 1981; von Foerster, 1984), biology and neurobiology (Maturana and Varela, 1990) and the calculus of form (Spencer Brown, 1969), Luhmann concludes that complex systems -including biological, psychological, and social ones- are autopoietic and operationally closed, meaning that their operations can only be determined by other operations of the same systems on the basis of the conditionings they set for future operations. Thus, Luhmann’s famous statement that individuals do not ‘communicate’, only communications communicate: while individuals can perform communicative utterances (e.g., by speaking), thus creating communicative opportunities, we may only observe that a “communication” is occurring when a new utterance occurs attributing a meaning to the previous one: that is, communication only occurs by referring back to previous communications and when it is referred to in future ones. The meaning of communication cannot be determined by the individuals taking part in it, nor the physical environment around it, but only by the context of communication in which it takes place. This does not mean, of course, that they are irrelevant for communication: communication usually takes place through utterances that are performed by “people”; that is, that cannot usually take place without the participation of minds, brains, and bodies. Even physical conditions of the environment can have an influence, for instance, very little communication can be achieved if the lack of an atmosphere impedes the transfer of sound; while the availability and functioning of technical apparatus may be needed to ensure that communication can be diffused quickly and broadly enough for all that need to take part on communication to be able to do so.

What Luhmann means by this is rather that all these non-communicative conditions can do is foreclose or trigger communicative opportunities, by they cannot give ‘meaning’ to communication. Meaning is only given recursively to communication by subsequent communications, that is, self referentially (or autopoietically, to use Luhmann’s terminology).

It must be noted that this self-enclosing of society is not a ‘choice’, or a mere contingency: on the contrary, communication can ‘only’ occur selectively: the world itself is too complicated, made up of too many possible elements, processes and relationship for communication to possibly be able to try and represent all of them, and even less hope that a subsequent communication can attribute it meaning and reproduce it. So, communication -and thus society- *requires* that the system close I tself up from the excessive complexity of the external environment, so it can recreate within itself a more manageable, self-constructed complexity. Importantly, this also applies to the *internal* complexity of society: society tends to differentiate itself in simpler, more manageable systems, each of which needs to rely on very unlikely coordination among selections that happen at the same time in other systems, and which it cannot control.

This has become even more evident in modern society, which has progressively abandoned a form of differentiation based on relatively enclosed ‘strata’ or ‘classes’ in favor of ‘functional’ systems (economics, politics, science…), each of which differentiates themselves from the others due to the specific ‘function’ it plays (e.g., to ensure provision in the face of scarce resources in the case of the economics, to take collectively binding decisions for politics, to produce ‘true’ knowledge for science, and so on…). Similarly, each has developed a unique communicative coding, like money, power, or truth (Luhmann, 1993, 2007). And while functional differentiation allows modern society to process an increased complexity and internal diversity, at the same time it reduces its capacity to resonate to uncoded events (such as biophysical changes, the individual needs of people, etc.) and to coordinate the different functional logics in which it is articulated: even ecological problems will necessarily be processed according to the rationality of each system. Therefore, the environment may make sense for the economic system only through costs and prices, for science only through evidence and theories, for politics only through its impact on the elections and on public opinion, and for the juridical system only through law and tribunals (Luhmann, 1989, 2012).

This hides a paradox, for in closing itself to the complexity of the outside world, communication must assume -without being able to control- the existence of a very complex (and unlikely) set of conditions: not only a livable world, but a world in which a very complex and unlikely form of life such as humans can survive and thrive, where minds can learn to grasp the sufficient degree of linguistic and semantic complexity to take part in communication, and where technical apparatus exist which ensure that communication can be diffused quickly and broadly enough for all that need to take part on communication to be able to do so, and so on. Moreover, the more complex the society becomes, the more autonomous and specialized its systems grow into, the more they ‘need’ each other to perform operations they have renounced to maintain or even to understand, a fact which can be stated simply by saying that the more autonomy a system gains, *the more dependence it experiences from all the rest* (Willke, 1987). From the point of view of sustainability, this paradox turns into a looming tragedy: it may well be that, the more society increased its internal differentiation and complexity, the more it risks becoming incapable to adapt to the complexity of its own environment -that is, the more it becomes unsustainable. In this context, understanding the conditions generated by these social structures seems essential to address the transformations and transitions that are required in the context of the ecological crisis. In addition, this begs the question of what possible mechanisms of governance can overcome the growing differentiation of society to promote a coordinated self-orientation of social systems towards the challenge posed by sustainability.

### Governing and transforming couplings: towards an integrated socio-techno ecological framework

One crucial aspect to consider when looking at socio-ecological and socio-technical systems theories is what do they specifically understand by system. As discussed above, the guiding idea in both cases was to overcome the traditional way in which traditional epistemologies have tended to separate the social and the material worlds: society, on the one hand, was seen as the domain of deliberate action (and interaction), of the subject (and the spirit), of decision (and freedom), of communication, cognition, and meaning; while natural or technical processes were seen as rather deterministic, deprived of an inherent meaning (or value), disconnected from the social world if not for relationship of dependence or domination. On the contrary, by talking of socio-ecological and socio-technical systems, these theories explicitly try to overcome this divide, emphasizing on how social, ecological, and technical processes feedback and interpenetrate each other, and should be seen as a one, an integrated whole, more than the sum of its parts (i.e., with emergent properties), and on top of that a whole able to some degree of self-organization: in other words, a complex adaptive system (Urquiza Gómez and Cadenas, 2015; Markard et al., 2020; Valencia et al., 2021). However, both traditions tend to display a rather lack of reflection on the nature and scope of these interactions, added to a superficial analysis of the social structures involved in the couplings.

Speaking of socio-ecological or socio-technical systems does not mean that the difference between domains stops existing, but rather, that one chooses to focus on how different kinds of processes, themselves different in nature and behavior, couple with one another. Moreover, if it makes sense to pursue an integrated look upon social and ecological processes, and social and technical ones, on the other, there is no reason to not further integrated the observation towards accounting for a couple of socio technical-ecological processes. But what is, then, the nature of these couplings, how may we identify and analyze them, and how can this inform deliberate attempts at promoting and governing transformations in socio-techno-ecological systems?

As anticipated, to help bring light to this, we plan to turn to Niklas Luhmann’s Social Systems Theory. Of course, this begs the question: what may a theory that explicitly struggles to make communication -and thus society- autonomous from its environment have to teach to approaches that, on the contrary, seem to be trying to overcome the boundaries separating society from the natural or technical worlds? The answer is that, specifically because Social Systems Theory assume that society and its environments are differentiated, and in fact, significantly limits how they may be coupled, it can be used to refine our understanding of these couplings and how they can be treated by both analytical and governance efforts concerning themselves with socio-techno-ecological transformations.

In fact, as already introduced before, it is possible to point a way a luhmannian re-framing of socio-ecological and socio-technical systems which at once acknowledges their ontological and epistemological disparities, but also offers a conceptual construct which illuminates a way to bridge the apparent dychotomy between realism and constructivism *from* a symbolic-communicative perspective.

A first pointer in this direction comes from Knoblauch and Pfadenhauer (2023), who argue that the dichotomy between realism and constructivism can be addressed through a Luhmannian reinterpretation of communicative constructivism. These authors critically reflect on how these two theoretical currents can complement and enrich each other rather than being seen as antagonistic. Critical realism focuses on the importance of understanding social reality as independent of individual interpretations and recognizing the existence of objective social structures. On the other hand, constructivism focuses on how social interpretations and constructions shape our understanding of the world. However, communication plays a fundamental role in the relationships between these two perspectives. Communicative processes reflect reality and actively construct it through social interaction and the negotiation of meanings. Likewise, constructivism can enrich critical realism by emphasizing the importance of intersubjectivity and the shared construction of meanings in forming social reality.

Similarly, Eberle reflects that the concept of realism adopted by communicative constructivism is broader and more elaborate than other approaches, such as ethnomethodology. In this sense, it recognizes the existence of an external reality. However, it emphasizes that this reality is fundamentally social and constructed through socialization, institutionalization, legitimation, power, and digitization and mediatization in contemporary society. Social reality is understood as a set of institutional structures and communicative processes that must be continuously reproduced to maintain stability but can also change over time. This reality materializes as a certain factuality independent of individual will and is shared with others through reciprocity. Furthermore, social reality is not limited to what individual actors perceive and construct in a social situation but also includes all the communicative actions of other actors and all the objectifications that are out of their sight, implying an impact and power behind their backs. Therefore, communicative constructivism adopts a concept of realism that recognizes the existence of an ‘objective’ reality (both in a material-physical sense: there is a world out there; and a social-cultural way: there is a society with structures that exist around us) but also emphasizes its socially embedded nature and its construction through intersubjective and communicative processes (Eberle, 2023).

By recognizing the interaction between the factuality of social structures and the intersubjective construction of meanings, the dichotomy between realism and constructivism can be transcended. This communicative approach offers a comprehensive and nuanced view of social reality, encompassing its objective aspects and socially constructed dimensions and processes of social transformation. The key principles underscore the importance of understanding the social construction of reality as a relational process involving communication, materiality, and corporeality, as well as the interaction between subjects in creating and negotiating meanings.

In fact, we always need to remember that in saying that the (communicative) society is aytonomous from the (material) environment, Luhmann does not at all mean to say that it is independent, or that the environment does not matter for the social. Quite on the contrary, the non-social environment does play a role (and a very important one) in Luhmann’s rendering: as explained before, through the difference between autonomy and independence. While the environment cannot directly determine communication, it can exclude communicative possibilities, and trigger disturbances in the system. And the more autonomous a system becomes, the more interdependent it grows. But then, it will be system structures that will determine which external disturbances will trigger irritations within the system and thus push a reaction on its part and what this reaction will be. To process the difference between autonomy and interdependence, systems enter into what Luhmann calls structural couplings: forms of selective openness of one system to specific disturbances of another (while it ignores most of the other disturbances as blank noise).

In a previous work (Billi et al., 2023) we referred to structural couplings as second order couplings to distinguish them from operational or first-order couplings. While the latter is given by the structural selection performed within each system concerning its operations within the boundaries of its autonomous self-reproduction, structural couplings work at the interface between systems, making it easier for them to reciprocally irritate each other since it makes it more likely that their operations -which exist in the environment of the other system- are taken by the latter as relevant instead of pure noise. This can work within the limits of society -between its partial systems or at the interface between society and its natural and technical environment. An example of the first are contracts (coupling law and economics), degrees (coupling economics and education) consultancies (coupling science and politics), etc. Examples of the latter are language (coupling individual consciences and social systems), bodily senses (coupling a body with its environment), and corresponding sensors (coupling technical systems with the physical environment), and so on. All of these are selective—they only process a limited degree of their environment’s complexity to make it available to the system-; all of them act as ‘translator devices’ to help different systems process their interdependence despite their reciprocal autonomy.

Based on this, we can understand that what we initially called a socio-techno-ecological system is not, in fact, a system, but rather a system-of-systems, made up of multiple structurally coupled ecological, technical and social systems. Each of these has its own structures and operational couplings, and is autonomous from the rest, but in its interdependence has evolved specific mechanisms of structural coupling. Thanks to these, together they may act as a larger system, which adopts emerging properties and behaviors, autonomous from its constituting systems, although strongly dependent on them. Importantly, ecological and technical systems are mostly made up of interchanges of matter and energy, and thus their coupling also exists at this level, i.e., associated with the use of resources or elimination of excesses. The social systems, however, are mostly semiotic, and immaterial, and thus their coupling with ecological and technical systems is mediated by meaning, and thus my language, semantics, and so on. In any case, since many of these couplings tend to require at least a certain degree of physical interaction, in the past we have call this secondarily coupled system-of systems territory, defining territory as spatially delimited and multi-scalar plexa of ecosystemic, technical and socio-cultural interrelations, processes and dynamics jointly determining the definition and satisfaction of water and energy needs (Urquiza and Billi, 2020).

Other examples of second-order cand how they may operate in constituting a socio technical system, such as, e.g., heterophonyc organizaztions, boundary objects, or interaction systems, are described in Billi et al. (2023). the case in many communication forms, now often non requiring any physical interaction, but can also affect technical and even natural systems: the concept of telecoupling, for instance, is used in the ecological literature to refer to the way in which environmental systems can be connected across far distances, mostly by technological or governance processes (Liu et al., 2018).

Now, it must be understood that most of these coupling emerge in a spontaneous (or evolutionary) way: one thing that both socio-ecological, socio-technical and social systems theory have in common is that they rely on a quasi-evolutionary understanding of systemic change (Geels, 2011; Urquiza Gómez and Cadenas, 2015; Büscher, 2022; Mascareño, 2022), which can be summarized like this: change is the product of the iterative accumulation of variations (patterns of operation defying a system’s dominating structures, also called regimes or attractors depending on the literature) which gradually build up a critical mass until they rival said structures and, if the conditions are adequate (if the context or landscape in which the regime operates are favorable, if the regime itself has become too rigid and unresilient) they can emerge to rival and even substitute the dominating structure, producing a regime shift in the system: so an ecosystem changes from a state of equilibrium to another, a new technical paradigm is introduced, or a political system is thrown down. In this understanding, structural couplings do not ‘produce’ change in the system, but they can trigger it, they can provide favorable conditions, can gradually erode the resilience of existing structures while empowering new ones. So they do influence change, and at the same time, they can themselves be changed: new structural couplings can emerge, while others may disappear or wane. We propose that is what may be called a transformation: when not only individual systems switch to other pathways, but when their very way of relating with other systems (e.g., society’s way of relating with nature *and* technology) is drastically changed, so that new reciprocal disturbances become relevant, new ways of influencing -or respecting- one another and so on.

Now, however, while transformations can occur -and have occurred- spontaneously, again as the product of multi-system, coupled evolution, it can also, under certain circumstances, be induced, or rather ‘organized’, and that is, in fact, the deliberate intention behind much socio-ecological and socio-technical systems literature: not just understanding how different ecological, social and technical systems exist and couple one with the other (not just understanding their interdependence) but also and specifically try to intervene or steer it, towards more desirable scenarios. That is what adaptive governance and transition management are about, and that is where we turn next.

### Governance and third-order couplings

Governance is a very polysemic concept and has received multiple and evolving definitions in and out of the social sciences (Kooiman, 2003; Meuleman, 2008; Voß et al., 2009). Within social systems theory, governance is usually considered a contemporary form of regulation (Bora, 2014), where the latter refers to the future oriented and deliberate attempt to reduce a difference’between an observed state of the world and desirable one (Luhmann, 1997): in the case at hand, it may apply to reduce current levels of pollution or greenhouse gas emissions, reduce future risks associated to climate change or promote more sustainable lifestyles (in the latter case, what is reduced is the gap between the ideal of a sustainable lifestyle and the current practice). Regulation, also called steering or self orientation can occur in several ways, and in fact organizations (state institutions, private corporations, organized communities and so on) mostly exist to counterbalance the tendency of modern functional differentiation to open contingency to virtually any possible state of the world, to instead selectively advance towards determined alternatives at the expense of others (Luhmann, 2018).

Governance is, for social systems theory, a contemporary phenomenon born from the gradual increase in the complexity of society enhancing the need to balance the autonomy (of autopoietic social systems such as economics, politics, law, science, and all the organizations that operate in and in-between these) and the interdependence that comes hand-in-hand with it (Willke, 1987). We can thus say that governance is all about balancing the factuality of autonomy with the need for coherence – a coherence required to manage the interdependencies that do exist between systems (Billi et al., 2020), a fact that is also acknowledged, with slightly different terminologies, by socio technical (Voss et al., 2009) and socio-ecological scholars (Cosens et al., 2018).

Our proposal, building on the conceptualization we were advancing in the previous section, is that what governance does is build third-order couplings: if first-order coupling is the structures that connect operations within each system, and second-order couplings are interfaces that connect the structures selectin operations between systems, then third-order couplings are mechanisms that connect different structural couplings to deliberately attempt to organize them, and by doing that steer the underlying systems in the desired direction. Third-order couplings can operate at different levels: in interactions, through deliberative systems (Willke, 1987) or hybrid fora (Callon et al., 2009); in organizations, through boundary objects and translating mechanisms working (Urquiza et al., 2018a,b; La Cour and Højlund, 2017; Kjaer, 2018); at the level of self-descriptions and norms (Kooiman et al., 2008; La Cour and Åkerstrøm Andersen, 2016; La Cour and Højlund, 2017; Pahl-Wostl, 2019). They are thus a correspondent of what literature sometimes calls meta-governance ^5^(Kooiman and Jentoft, 2009; Meuleman, 2018). The task that these mechanisms need to play can also be usefully detailed by reading it through what Luhmann (1999) would call the ‘three dimensions of meaning’ (in this case, the meaning of the transformation that governance is attempting to entail):

- The factual framing of the system and its transformation problem: multiple possible ‘delimitations’ both concerning the problem that governance seeks to address (e.g., which scales, sectors, processes, systems are object of governance) and concerning what constitutes ‘governance’ itself (institutions, actors, instruments, decisions, etc.) that seeks to coordinate around said problem. It can be synthesized in terms of what actions, subjects, times and spaces are sought to be transformed or governed, and through what actions, processes, institutions, instruments, etc. Thus, the challenge in this dimension is balancing Universality (rules and instruments consistent with each other and applicable in an orderly manner to different contexts) and Specialty (adaptation of said rules and instruments to the particularities of different sectors, scales or systems).
- The social mediation between the stakeholder perspectives around the transition and its governance. Multiple and differentiated perspectives and rationalities exist regarding the problem that governance seeks to respond to, the result it seeks to achieve, the best ways to resolve it, and also the very way of making decisions in this regard (who decides, for the benefit of ‘who makes the decision, to whom and how they are accountable for those decisions, etc.). It is reinforced in the presence of inequalities in the impacts or distribution of costs and benefits, dilemmas (trade-offs) associated with decisions that benefit some group or area to the detriment of others, and controversies about the definition of the problem or its solutions. The balance challenge, then, here takes the form of a tension between Coherence (or perhaps it would be more accurate to say ‘control’, in the form of the capacity of certain decisions to effectively influence others) and autonomy (and diversity, of the perspectives and rationalities to which these decisions refer).
- The temporal observation of the transition and its different and overlapping timelines: multiple possible purposes or trajectories exist towards which governance could push the solution of the objective problem, and associated with that, different ways of defining the goal of governance and evaluating its success or failure. It is also related to the existence of multiple time horizons (short and long term, reactive and proactive, etc.) and how changes and maintenance are measured throughout them. Balance then here refers to the dichotomy between Orientation (in the sense of the ability to generate anticipated or desired effects on the future performance of target systems) and flexibility (which involves being able to adapt to changing conditions in that these systems operate).

In the next section, we explore how to build ‘third order’ linkages, and by doing so, we can push the underlying systems in the desired direction (governance) to achieve sustainable change. In this process, the design of the transitions can help to understand how to activate the interactions through deliberative systems and translation mechanisms in charge of achieving a meta-governance direction (steer) in the transformation processes.

### Moving to sustainability transition in action

Transition Design (Irwin, 2015) is an emerging approach seeking to facilitate societal transition processes by supporting, connecting, and developing interventions to intentionally change values, technologies, social practices, and infrastructures while reshaping interactions between socio technical and socio-ecological systems. Transition Design’s (TD) tools and practices amalgamate theory and mindsets across various fields and knowledge systems, and promote collaborative spaces of practice, learning and experimentation. Its reflective and practical approach to dealing with systemic issues offers a way to envision and enact alternative collective ways of being and knowing, and thoroughly embraces the concept of the pluriverse—a world where many worlds fit (Schön, 1984; De la Cadena and Blaser, 2018; Escobar, 2018). Its focus on deliberation, experimentation and context specificity demand that actors are encouraged to question and jointly reframe their values (Schön, 1984) in a process of collective and self-transformation.

The literature describing this design approach proposes three main iterative steps: problem framing and reframing, the generation of collective visions, and the proposition of transition pathways which include the generation of potential synergistic interventions (Öztekin and Gaziulusoy, 2020; Kossoff and Irwin, 2021). In practice, these phases may be less clear cut or operationalized in different ways. Within the practice that is developed at SARAS Transition Lab (Zurbriggen and Lago, 2019; Zurbriggen and Juri, 2021), we prefer to conceptualize the process in four abstract phases. The first step implies developing a deliberative space with critical actors to frame the problem, understandingits interactions, historical evolution, root causes, and, in particular, dominant framings and narratives that impede a change process. A second step involves generating a change strategy by identifying a vision of desirable futures. Consequently, the goal is not to plan for a single future (near contingency) but to open alternative futures (open contingency): several paths can reach the desired state, and several plural paths can take place/to coexist.

However, the discussion must be structured to lead to a possible long term agenda or vision for change. A third step involves designing an intervention strategy: selecting a path from the current situation to the collectively constructed vision. This step challenges stakeholders to ask what they want to leave behind, what is not working, what we need to keep, and what innovations and new practices are required. This may include a series of material and symbolic interventions— known as ecologies of actions— to open opportunities that develop whole new narratives and lifestyles (Irwin et al., 2021) which unavoidably engage and challenge unsustainable values and paradigms. Finally, a fourth step involves reflective monitoring of the transition processes. Because transitions take place over long time horizons and their elements constantly change, an iterative, adaptive, and continuous learning attitude is required to observe and qualitatively evaluate change to correct it, considering preferred values, motivations, and visions of the future or paths of transition that each group normatively co-develops and recursively analyzes (Zurbriggen and Lago, 2019). Monitoring is based on creating a space for the negotiation of meanings (through understanding the contributions, accepting disagreements, or postponing stubborn responses, among other strategies) and the possibility of identifying common points where the main differences or misunderstandings are overcome (van Mierlo et al., 2020).

From this perspective, we can decompose the tasks and challenges of transition design along the three dimensions mentioned above: factual, temporal and social. For each, we will look at ways transition design can contribute to providing this function and the key challenges it has to face.

#### Factual dimension of the transition: framing the problem and the problem arena

As explained above, the factual dimension refers to the framing of the system and its transformation problem. Thus, this first step implied a recognition that the paths of governance are marked by many observations to which the actors draw attention. The actors make permanent observations about their environment, framed by different narratives and values, concerns, and impacts on the logic of the existing governance regime. Governance is an arena in which different worldviews collide, which can manifest in heated debates, but also in more subtle ways through processes by which different ways of knowing to compete for prominence and, as such, constitute themselves in power/knowledge relations. Specific ways of looking at and understanding a problem can lead to specific ways of managing or governing it. This is how social and spatial identities are built; stories about the past, present, and future are shared, or multiple interpretations of the issues are discussed and negotiated.

Understanding Luhmann’s perspective on communication and autopoiesis is valuable in designing transitions. In transition design, designing interventions involves improving and directing communications within social systems to foster positive changes. Furthermore, the focus on communication as the core of social systems highlights the importance of creating shared narratives and consensus that can facilitate the transition to new desired states in society.

Luhmann and Capra agree that social systems generate shared meanings and cultures through communication. In transition design, this means that it is essential to create and promote shared narratives and values that can guide and motivate actors toward the desired new reality. Communication strategies must be designed to build and reinforce these shared meanings. Moreover, Luhmann highlights the importance of second-order observation, that is, observing how systems observe themselves and other systems. In transition design, this involves being aware of how actors perceive and understand the change process and how these perceptions can influence the success of the transition. Designing mechanisms to reflect and adjust these perceptions can be key to a successful transition.

In this sense, as explained above, developing deliberative arenas around a problem (climate change) is necessary for improving communication and generating debates to promote action strategies (strategies, policies, programs). This process demands greater attention to the discursive dimension of how socio-material and environmental environments are observed and understood to ‘build’ systems as an assemblage between communicating systems through structural couplings with the consciousness systems of the actors responsible for the diachronic and differentiated transformation of the interventions in space that characterize them, helping to understand how communication process impact governance dynamics.

Within this process, the design of the transitions helps to develop new practical approaches to strengthen this dimension, such as working on the narratives and creating and collecting stories. That is, to work through integrating different voices with plural identities and perspectives and generate new interpretive frameworks for the problem. A critical task in designing transitions is helping to frame and reframe a problem collectively. By framing a public problem, the actors define what is problematic (selecting, emphasizing, and narrating), generating a basis for persuasion and a direction for action (governance) (Rein and Schön, 1994). Identifying the current frames allows us to detect elements that support a problematic situation and limit change processes. Reframing a problem collectively involves finding elements that promote new narratives, actions, and stories and visualizing problems and solutions from other frames. Reframing helps us to redefine a problem with new ways of understanding that can encourage new actors to participate in a new intervention in the system by creating a new common framework.

In this context, framing the problem is a beneficial approach to foster the building of the third-order system assemblage, as it creates a common reference that can be referred to as the identity of the emerging systems and their governance: the socio-ecological and socio-technical systems are enacted in this way, since this is what makes visible the second-order couplings which constitute, making them into steering problems which can then be available for future interventions and governance attempts.

#### Social dimension of the transition: promoting a reflexive dialogue

As discussed earlier, the social dimension of transition design is about developing a mediation between system stakeholders' different forms of knowledge and rationalities and their involvement in a co-creation process. This mediation between the multiple stakeholder perspectives around a problem impacts the evolution of governance and the change process. Nevertheless, these process tends to become black-boxedin governance studies.

In this process, it is critical to ask how we build the dialogue, whom we invite, exclude, and deal with this marginalization. Not all actors can be included, and we always put a limit on the system; judgments about whom we include and whom we do not include are critical to understanding how to address conflict and marginalization through a participatory design process are at the heart of systemic intervention (Midgley, 2023). Involving different perspectives also implies addressing power relations and accepting controversies, conflict, and disagreements as an inherent and necessary aspect of any transformation process since it determines the possible forms or courses of action adopted and discarded (and who and how they will impact or benefit). Nevertheless, not all conflicts are destructive. Conflicts can be constructive when they lead to dialogue and better mutual appreciation. New ways of thinking about challenges can be introduced by questioning existing assumptions and altering the boundaries of or between fields.

In effect, according to Luhmann, social meaning is constructed through communication and interactions between actors, which depend deeply, on the one hand, on the specific rationalities and systemic codes used for communication, and on the other, on the availability of structural coupling mechanisms and cross-cutting semantics which may help to translate from one rationality to the other In transition design, this dimension helps understanding the potential challenges and pathways needed to promote reflexivity, dialogue and collaboration between all rationalities involved. It is crucial to understand and reconcile the diverse perspectives, interests and expectations of stakeholders to build a overarching shared vision and identify of the deliberation system, and of the governance regime built around it, to ensure an inclusive, self-regulating and participatory transition process (Urquiza et al., 2018a,b).

Various transdisciplinary methods are valuable (e.g., brainstorming, mind mapping, actor mapping, and backcasting) in structuring the dialogue process and helping participants gain systemic insights into emerging and synergistic phenomena to frame and reframe the problem. The design of these reflexive spaces demands an openness to tools, which are unavoidably driven by different purposes (Bammer et al., 2020; Norström et al., 2020). Thus, this demands the creative design of methods (Midgley, 2023, p. 226), a synergistic combination of tools that address specific questions within the issues of concern. The flexibility in this creative adaptation eases and supports a more appropriate and contextual specificity in the process—with particular purposes, needs, and capacities that demand to be leveraged. The outcome of such an open, fluid, and reflective process can therefore emerge as a mutual learning space and process which encompasses the exploration and integration of helpful knowledge—either tacit or codified—for a deeper understanding of a problem, better decision making for change (Westberg and Polk, 2016). For example, it can include the arts-based methods (e.g., visual arts through drawing, painting, photography, animation, literary art, performing art, and other forms such as collage) that can help us expand our understanding, identify current narratives, and deconstruct the problem to find ways to reframe it. Reframing is fundamental to creating a new collective future vision considering the different actors perspectives involved in the participatory process. It generates a future vision and outlines potential actions that reflexively bring us closer to them.

#### Temporal dimension of the transition: creating a regulatory identity and a pathway for change

The temporal perspective involves, as explained above, coordinating the different and overlapping timelines involved in the transition, that is: the current trajectory, the future trajectory and the actions to drive the switch from one course to the other (Sharpe et al., 2016). From a governance perspective, steering the transition requires to ‘stabilize’ these distinctions (making them into identities) so that they can serve as a guide for the regulatory process and also as indicators of the success of such regulation (Luhmann, 1997).

In this context, Transition Design helps design systems interventions implemented at multiple scale levels over short, mid, and long time horizons. These interventions and the long-term vision and near-term milestones are connected along a transition pathway formed by backcasting from the future vision to the present. For this reason, the processes of transitions must create compelling communications and narratives explaining why action and observation are crucial to designing over long periods.

In this process, we can use different future studies methodologies to generate alternative visions articulated past, present, and future (e.g., Three horizons, Causal layered analysis). This brings us closer to Poma’s (2020) Andean Futurist Manifesto, which invites us to start reconfiguring frames and narratives to create futures by approaching the past. In the process of collective negotiation of the future worlds and making worlds are followed through the generation of assemblages (in the form of collages) that combine, compare, juxtapose, subvert, or transform disparate elements to create new meanings and therefore new possibles futures. In this process, we start by decomposing and harvesting from the distant past, filtering the present and seeds of possible futures, and mixing the past and present to compose a future vision expressed in images and as a textual narrative.

## Conclusion

The paper offered a transversal outlook to the literature on socioecological, sociotechnical systems and transition design, grounding them in the deep sociological knowledge embedded in Luhmann’s Social Systems Theory. From here, we introduced the concept of second-order coupling for a sociologically grounded understanding of the interactions making up socioecological and sociotechnical systems-heterogeneous and quasi-self-organized arrays of social, technical, and natural elements and processes. Their reciprocal interdependences bring these together, but these interdependencies only become relevant for the involved systems and, thus, may influence their behavior inasmuch they align with selective and emerging processes of structural couplings, which open up each system to account for some (but not all) interdependences with other systems. Thus, second-order couplings (structural couplings) mediate between the actual interdependencies between the systems (first-order or operational couplings) and the reaction or management that each system may have on them since they define what is considered relevant from the perspective of each system. However, the emergence of second-order couplings is left only to chance. In that case, it is unlikely that all the necessary interdependencies for the sustainability of the involved systems are considered. Third-order couplings, the very matter of governance, come in. They are the relationships between operations and structures mediated by a deliberate attempt to ensure coherence and coordination in the face of the autonomy and heterogeneity of socio-techno-ecological systems. We then observed what are the key challenges involved in this effort and how critical insights from the socioecological and sociotechnical literature can help overcome them.

This manuscript thus offers a deeper conceptual and methodological understanding of socio-techno-ecological couplings and systems in the context of sustainability transformation and offers insights into their governance. The need to understand and promote changes that include a technological and ecological dimension has led different disciplines to observe the links with society. In this context, incorporating a system understanding of the interactions linking up society, ecosystems, and technology in the context of transformations is a fundamental step to improve our understanding of these dynamics and advance new strategies to steer them towards more collectively desirable futures. Social structures generate multiple challenges for socio-technical and socioecological transformations, so understanding governance in its objective, social, and temporal dimensions makes navigating these challenges with better tools possible.

We believe this manuscript offers at least two interesting avenues for future work: the first, empirical, would point to applying these approaches to the practical study of concrete cases of transition and their governance, while the second, more theoretical, would require delving into more depth into the epistemological and even ontological differences and points of contact between Luhmann’s theory and other approaches reviewed in this paper, most notably the ones founded in critical realism, to possibly identify other points of articulation or comparison which may produce insight for the study of transitions. We hope we have motivated the reader at least to explore these intriguing possibilities.

## Data availability statement

The original contributions presented in the study are included in the article/supplementary material, further inquiries can be directed to the corresponding author.

## Author contributions

All authors listed have made a substantial, direct, and intellectual contribution to the work and approved it for publication.

## Footnotes

^1^In this text, Geels does indeed cite (functionalist) systems theory. However, he does not refer to Luhmann’s proposal but rather to the framework from Talcott Parson’s, on which Luhmann inspires the early foundations of his theory but also fundamentally departs from. Noticeably, Parsons was also a key influence for Jurgen Habermas, a critical theorist, thus displaying how the same original questions can them stem very different epistemological and ontological proposals -which can then be opposed but still usefully debate—if not necessarily agree-, as is clearly demonstrated by the decades-long theoretical struggle between the very Luhmann and Habermas.

^2^Luhmann himself has often described his work as a frame to observe frames.

^3^The keen reader may here wonder whether it is not hironic (or possibly, even as an epistemic fallacy) to wonder about the ‘reality’ of socio-technical or socio-ecological systems from the point of view of a theory which is usually taken to be struggling in the “de-ontologization of reality”) (Wan, 2011a,b). However, it should be noted that Luhmann’s stated purpose (e.g., in the prefacio to his flagship book, “Society of Society” (Luhmann, 2005) is less to de-ontologize reality and more to question the epistemological assumptions which, according to him, would be preventing sociological theory to explain (modern) society, and build a general theory of society that prescinds from those assumptions. There is no assumption about the fact that reality ‘exists’ or not at the heart of Luhmann’s thinking: in fact, what motivates him may be better described as skipping altogether that ontological question to focus on the epistemological question of how can society be known and made sense of (see Andersen, 2011). But at the same time, in depicting society as a self-referential construct that constantly expands and reproduces by “knowing itself” -or at least, by communicating about itself and making meaning—then the epistemological question on the conditions for observation also becomes a substantial question on the conditions for society self-observation, and by that token, the structure of society that Luhmann describes can be understood as the structure that society needs ‘if’ it must be able to communicate about itself and reproduce meaning within and for itself, which we experience daily that it does. While this is not a fully ontological statement, it is not purely epistemological either, but instead treads a fine line in the middle: brought to our problem at hand, the same approach may be applied to the ‘reality’ of socio-technical and socio-ecological systems. We are not trying to get to the ontological heart of what they are, but rather, to the question of how they may be make themselves into systems with meaning—and thus, systems that can be subject of governance.

^4^The keen reader may here wonder whether it is not hironic (or possibly, even as an epistemic fallacy) to wonder about the ‘reality’ of socio-technical or socio-ecological systems from the point of view of a theory which is usually taken to be struggling in the “de-ontologization of reality” (Wan, 2011a,b). However, it should be noted that Luhmann’s stated purpose (e.g., in the prefacio to his flagship book, “Society of Society”) (Luhmann, 2005). is less to de-ontologize reality and more to question the epistemological assumptions which, according to him, would be preventing sociological theory to explain (modern) society, and build a general theory of society that prescinds from those assumptions. There is no assumption about the fact that reality ‘exists’ or not at the heart of Luhmann’s thinking: in fact, what motivates him may be better described as skipping altogether that ontological question to focus on the epistemological question of how can society be known and made sense of see Andersen (2003). But at the same time, in depicting society as a self-referential construct that constantly expands and reproduces by “knowing itself”—or at least, by communicating about itself and making meaning - then the epistemological question on the conditions for observation also becomes a substantial question on the conditions for society self-observation, and by that token, the structure of society that Luhmann describes can be understood as the structure that society needs ‘if’ it must be able to communicate about itself and reproduce meaning within and for itself, which we experience daily that it does. While this is not a fully ontological statement, it is not purely epistemological either, but instead treads a fine line in the middle: brought to our problem at hand, the same approach may be applied to the ‘reality’ of socio-technical and socio-ecological systems. We are not trying to get to the ontological heart of what they are, but rather, to the question of how they may be make themselves into systems with meaning -and thus, systems that can be subject of governance.

^5^It could be discussed if these third-order couplings constitute themselves an emerging system. Most governance scholar, at least within the cybernetic and/or systemic thinking, agree that governance regimes have emerging properties and could thus be considered ‘systems’ in a broad sense. Whether they have the characteristics of operational closure and autopoiesis required to be systems in Luhmann’s stricter sense is more difficult to determine. Organizations are systems according to Luhmann, systems which process a particular kind of communications dubbed as ‘decisions’, and they do so in an autopoietic and operationally closed way. Governance regimes also process decisions and they are mostly self-referential in their operations, but they are a far less structured -and most notably, bounded—space, so that the concepts of operational closure and autopoiesis do not fully apply here. It could be argued that they are in a way quasi-systems or loose organizations. But this would required a deeper discussion which is best left for another manuscript. For this reason, in this text we have preferred the word governance ‘regimes’ instead than ‘systems’.
